# Anthrax prophylaxis: recent advances and future directions

**DOI:** 10.3389/fmicb.2015.01009

**Published:** 2015-09-24

**Authors:** E. Diane Williamson, Edward Hugh Dyson

**Affiliations:** Defence Science and Technology LaboratoryPorton Down, Salisbury, UK

**Keywords:** pathogenesis, anthrax, toxemia, prophylaxis, therapy, adenylate cyclase, MAP kinase

## Abstract

Anthrax is a serious, potentially fatal disease that can present in four distinct clinical patterns depending on the route of infection (cutaneous, gastrointestinal, pneumonic, or injectional); effective strategies for prophylaxis and therapy are therefore required. This review addresses the complex mechanisms of pathogenesis employed by the bacterium and describes how, as understanding of these has developed over many years, so too have current strategies for vaccination and therapy. It covers the clinical and veterinary use of live attenuated strains of anthrax and the subsequent identification of protein sub-units for incorporation into vaccines, as well as combinations of protein sub-units with spore or other components. It also addresses the application of these vaccines for conventional prophylactic use, as well as post-exposure use in conjunction with antibiotics. It describes the licensed acellular vaccines AVA and AVP and discusses the prospects for a next generation of recombinant sub-unit vaccines for anthrax, balancing the regulatory requirement and current drive for highly defined vaccines, against the risk of losing the “danger” signals required to induce protective immunity in the vaccinee. It considers novel approaches to reduce time to immunity by means of combining, for example, dendritic cell vaccination with conventional approaches and considers current opportunities for the immunotherapy of anthrax.

## Introduction

### Anthrax: The disease

Anthrax is one of seven globally-neglected diseases, which are both zoonotic and endemic, according to the WHO (http://www.who.int/zoonoses/neglected_zoonotic_diseases/en/). Anthrax is primarily a disease of herbivores that become infected through contact with soil contaminated with persistent bacterial spores. Humans are susceptible to infection through contact with infected animals or animal products contaminated with bacterial spores. Anthrax is caused by the Gram-positive sporulating bacterium *Bacillus anthracis*, as first identified by Robert Koch (Koch, 1876). In spore form, the bacterium can exist in the environment for centuries (World Health Organization, Food and Agriculture Organization of the United Nations, World Organization for Animal Health, 2008), surviving desiccation and thermal extremes. Once the bacterial spore gains entry to a mammalian host, it is taken up by phagocytic cells and transported to the draining lymph nodes (LNs) where host cells undergo apoptosis and the bacteria germinate into vegetative cells able to secrete the tri-partite complex of anthrax toxins (reviewed in Abrami et al., 2005).

Depending on the route of exposure, natural anthrax infection in man can take three forms: cutaneous, gastro-intestinal or most seriously, pneumonic anthrax (Plotkin and Grabenstein, 2008). Recently a fourth form, injectional anthrax, due to the intake of contaminated heroin, has been documented (Berger et al., 2014). Following inhalational exposure to *B. anthracis*, the spores accumulate in the lung alveoli and are then engulfed by migratory cells (macrophages, dendritic cells or neutrophils) which traffic to the mediastinal and peribronchial LNs. There the bacteria multiply, causing hemorrhagic mediastinitis, and subsequently spread throughout the body via the bloodstream causing symptoms of fever, malaise, fatigue, and mild chest discomfort, progressing to severe respiratory distress with shock, cyanosis, and death (reviewed in Guichard et al., 2011). The infection is characterized by a prodromal phase, which may be asymptomatic, followed by a sudden onset fulminant phase leading to an abrupt deterioration into respiratory distress, sepsis, shock and death. If undetected and untreated, the mortality rate can be very high. In 1979, an outbreak of anthrax in Sverdlovsk (former USSR), resulted in an 85% mortality rate in exposed people (Meselson et al., 1994). However, the prompt and improved intensive care of victims of the 2001 postal attacks resulted in a mortality of 45% (Jernigan, 2001; Jernigan et al., 2002; Perkins et al., 2002), whilst therapy of anthrax infection in drug users has been successful (Berger et al., 2014).

### Current and future vaccines for anthrax

There is an ongoing effort to produce better defined vaccines for anthrax with contemporary formulations and presentations. This has been based on an evolving understanding of the complex pathogenesis of anthrax infection and the fact that it is insufficient to merely reduce the bacteremia with antibiotic therapy, since beyond a certain tipping point, the toxemia of anthrax is fatal (Guichard et al., 2011). Vaccines comprising live attenuated strains of *B. anthracis*, such as the STI strain, have been used in the former USSR to vaccinate people and a veterinary vaccine comprising the Sterne strain is still used globally to vaccinate cattle (Turnbull, 1991). Licensed human vaccines for anthrax, comprising filtered supernatants from bacterial cell cultures, have been in use for the last 60 year or more (Plotkin and Grabenstein, 2008). These vaccines are termed Anthrax Vaccine Absorbed (AVA) or Anthrax Vaccine Precipitated (AVP) in the USA and UK, respectively, since the vaccine is either absorbed to, or precipitated with, alum salts (Anthrax Vaccine Adsorbed, 1965; Hepburn et al., 2007; Wright et al., 2010). The AVA and AVP vaccines, made by batch culture of pX01^+^/pX02^−^ *B. anthracis* strains, comprise predominantly PA with trace quantities of various other bacterial derived components. AVA contains traces of LF but is virtually free of EF, while AVP contains some LF and traces of EF. However, due to the method of production, the relative concentrations of these proteins in consecutive batches can vary and in the UK, AVP is produced within biocontainment.

To achieve a more defined vaccine, the recombinant expression of PA and/or LF has been pursued and a number of candidate rPA vaccines are currently in development and in clinical trials for safety (Plotkin and Grabenstein, 2008). Additionally formulations comprising spore coat proteins in combination with PA have been proposed (Brossier et al., 2002). In subsequent sections, the virulence and pathogenesis of *B. anthracis* is reviewed, in order to place in context the effort toward next generation vaccines for anthrax.

### Virulence factors from *B. anthracis*

*B. anthracis* expresses a number of virulence factors. The poly-D-glutamic acid (PGA) capsule, encoded by the pXO2 plasmid, is a major virulence determinant; it ensures environmental survival of the spore and *in vivo* disguises the bacterium from immune surveillance and protects it from phagocytosis (Jang et al., 2011). PGA is weakly immunogenic and *in vitro* the capsule can activate caspase1 and induce IL1β release from THP1 and human monocyte- derived dendritic cells. PGA was observed to associate with LT in the blood of infected animals (Ezzell et al., 2009) and further research showed that PGA enhanced the cytotoxicity of LT for murine cells *in vitro* and the toxemia of terminal anthrax *in vivo* (Jang et al., 2011). During infection, PGA may also target the bacteria to enter the vascular endothelial cell (VEC) wall, from where they can secrete toxins (Piris-Gimenez et al., 2009).

An S-layer protein, BslA, is encoded by the pXO1 plasmid and is another virulence factor; it promotes adhesion to mammalian cells including VECs. A pore-forming toxin, anthrolysin, is also secreted by *B. anthracis* and activates the Toll-like receptor 4 (TLR4) on macropahges, inducing apoptosis. Anthrolysin acts in concert with EF and LF to enhance toxemia (Guichard et al., 2011).

*B. anthracis* also secretes proteases which contribute to its virulence by reducing cellular barrier function thereby promoting vascular leakage, and also by triggering the host coagulation cascade. In this context, DIC and profound thrombocytopenia appear to be pre-mortem signs in injectional anthrax—possibly markers of the tipping point (Berger et al., 2014).

All of these virulence factors contribute to pathogenesis, and may interact with the toxins secreted by the bacterium. However, although the bacteremia can be treated with antibiotics, if the toxemia progresses beyond a tipping point, it will ultimately prove fatal.

### Pathogenesis of anthrax infection

During infection, *B. anthracis* spores germinate and release three proteins, termed Protective Antigen (PA), Lethal Factor (LF), and Edema Factor (EF; Smith, 2002). Both LF and EF are enzymes: LF is a zinc metalloprotease that cleaves the N-terminus of several mitogen-activated protein kinase kinases (MAPKKs) or MAP/ERK kinases (MEKs) and EF is an adenylate cyclase. PA is the cell-binding component of a binary complex with LF (to form lethal toxin, LT) or with EF (to form edema toxin, ET). LT and ET are potent toxins (Abrami et al., 2005).

**Figure 1:**
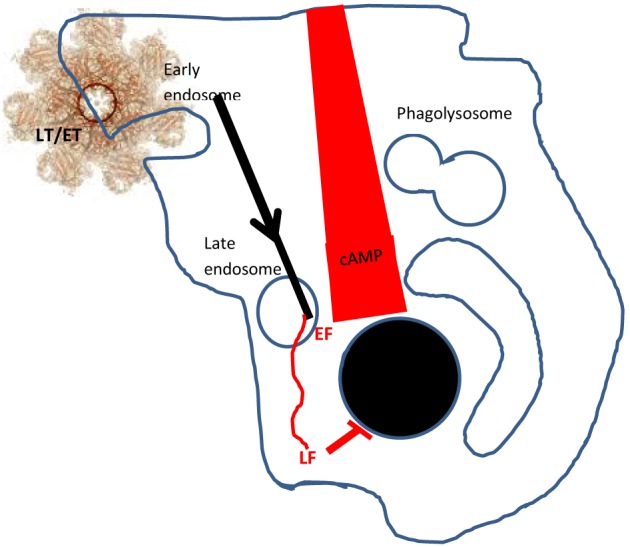
**Delivery of LF and EF into the host cell**. PA 83 binds to cell surface receptors and is subsequently cleaved and oligomerises to form a heptamer (PA^7mer^). LF and EF can bind to the PA^7mer^ to form lethal toxin (LT) or edema toxin (ET) which associate with lipid rafts. These complexes are endocytosed (in clathrin-coated pits, facilitated by LRP6) and enter early endosomes. Subsequently, LT/ET are conveyed in vesicles to late perinuclear endosomes. The PA^7mer^ forms a pore in the vesicle luminal wall, releasing EF to the membrane and LF to the cytosol. EF creates a gradient of cAMP emanating from the nucleus to the cell wall, whilst LF cleaves the MAP/ERK kinase (MEK) substrate to inhibit nuclear protein synthesis. Glossary: MAPKKs, mitogen-activated protein kinase kinases; ERKK, extracellular-signal-regulated kinases; MEK, MAP/ERK kinases.

Much is now known about the process of host cell-binding by PA prior to endocytosis of either of these toxic complexes (Abrami et al., 2005). The full-length (83 kDa) PA binds to one or more host cell receptors: Capillary Morphogenesis factor 2 (CMG2), Trans-endothelial membrane receptor 8 (TEM8), or β1-integrin (Martchenko et al., 2010). It then undergoes furin cleavage to release a 20 kDa N-terminal fragment, leaving a 63 kDa truncated protein (PA_63_) which is subject to a structural rearrangement with heptamerisation, so that domain 4 is in contact with the host cell receptor. In the process, binding sites for either three molecules of LF or EF are exposed on units of the PA heptamer (a maximum of nine toxic complexes are generated per PA ^7mer^) and the latter associates with lipid rafts, mediated by the lipoprotein-receptor-related protein 6 (LRP6; Wei et al., 2006). Subsequently, the toxic complex undergoes clathrin-mediated endocytosis (Abrami et al., 2010) and enters early endosomes where it is incorporated into intraluminal vesicles (Figure 1). Acidification of the endosome allows insertion of the PA^7mer^ into the membrane of the intraluminal vesicles as well as the unfolding and release of LF and EF through the channel of the PA^7mer^, into the lumen of the vesicle. Subsequently, both LF and EF undergo microtubular transport through the lumen of the vesicles to late perinuclear endosomes. Here, the vesicles can undergo back fusion with the limiting membrane so that LF is released into the perinuclear cytoplasm (Liu et al., 2003; Abrami et al., 2006; van der Goot and Young, 2009) where it cleaves MAPKKs to disrupt phosphorylation and transcription in the nucleus, ultimately preventing protein synthesis and causing cell death. EF, a calcium and calmodulin-dependent adenylate cyclase, remains bound to late perinuclear endosomes, where it causes a rapid increase in perinuclear cAMP resulting in cellular, tissue and ultimately organ oedema (Tang and Guo, 2009).

### Evasion and antagonism of the host immune response

It is hypothesized that LF and EF have evolved to use this cellular entry pathway to evade exposure to lysosomal proteases and the host immune response (van der Goot and Young, 2009). Both LF and EF suppress host cytokine secretion (Tournier et al., 2005) and weaken vascular endothelial barriers by downregulating vascular cadherin, important in cell-cell adhesion (Guichard et al., 2010). This effect is thought to contribute to the vascular leakage typical of systemic anthrax (Moayeri and Leppla, 2004).

**Figure 2:**
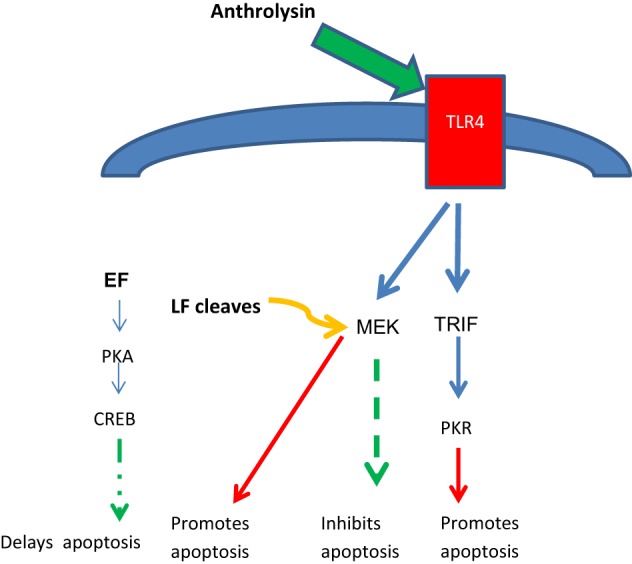
**Opposing effects of anthrolysin, LF and EF on host cell apoptosis**. Anthrolysin secreted by *B. anthracis* binds surface TLR4 receptors with downstream signaling through either TRIF and PKR to promote apoptosis, or through MEK to inhibit apoptosis; the latter inhibitory effect is opposed by LF cleaving MEK, thus promoting apoptosis. EF signals through the PKA and CREB pathway which has benefit in delaying apoptosis until the phagocytic host cell reaches the lymph node, an optimal niche for germination with further toxin release. Glossary: PKR, protein kinase regulated by RNA; PKA protein kinase A; CREB, cAMP response-element-binding protein; TRIF, TIR-domain-containing adapter-inducing interferon-β.

ET appears to have co-opted a host signaling pathway to facilitate the transport of bacteria from the lung to LNs. It is hypothesized that ET mimics the anti-inflammatory action of G-protein coupled receptors (GPCRs) to induce macrophage migration, ultimately delaying apoptosis and increasing the delivery of bacteria by macrophages to the LNs (Abrami et al., 2005, 2006; Tang and Guo, 2009; Guichard et al., 2011). Intracellularly, EF and LF appear to act synergistically to delay apoptosis. The pore-forming toxin anthrolysin binds TLR4 receptors, providing conflicting signals to induce apoptosis (via PKR) or to inhibit via MEK. However, by cleaving MEK's, LF blocks this inhibitory and protective signal, shifting the balance of TLR4 signaling toward apoptosis. EF counters this effect by signaling through the PKA and CREB pathways, protecting the macrophage during its migration to the LN where apoptosis occurs to release bacteria and spores (Figure 2), although this effect is not definitive and in some models ET has been proposed to inhibit macrophage migration (Guichard et al., 2011).

Observed differences in the sensitivity of rodents to the effects of anthrax toxins *in vivo* have led to further study of *Nlrp1b*, a component of the inflammasome pathway which mediates cell death in LT-sensitive macrophages through a rapid non-apoptotic mechanism termed pyroptosis (Guichard et al., 2011). *In vivo* susceptibility of LT-sensitive rodents did not however correlate with macrophage sensitivity *in vitro*, suggesting that premature killing of macrophages and inflammasone activation in *Nlrp1b*-sensitive strains may lead to IL1-mediated neutrophil influx and bacterial killing, thus reducing bacterial dissemination and promoting survival. However, human and non-human primate macrophages have been shown to be resistant to LT pyroptosis (Ribot et al., 2006), undergoing the slower process of apoptosis, and so the relevance of the *Nlrp1b* inflammasome pathway in anthrax infection in humans is uncertain.

There have been many reports, some conflicting, on the effect of anthrax toxins on host cell cytokines and the induction of immune signals and bactericidal factors. Overall, LF and EF both suppress host cell cytokine signaling and cell surface activation markers to achieve suppression of the host immune response (Moayeri and Leppla, 2004; Tournier et al., 2009).

### End-stage toxemia

The current paradigm is that in fulminant anthrax infection, anthrax toxins kill the host by direct effects on the cardiovascular system (Guichard et al., 2011). ET and LT decrease the vascular barrier integrity of VECs; ET and LT cause cardiovascular dysfunction with ET causing hypotension by increasing vascular permeability and vasodilation, whilst LT acts directly on the heart to compromise structure and performance. Overall, the combined effects of the toxins is to break down the barrier between the vasculature and the tissues, causing systemic effects with fatal outcome, leading to disseminated intravascular coagulation (DIC), profound thrombocytopenia, respiratory shock and cardiac failure (Berger et al., 2014). There may also be CNS involvement as bacteria are disseminated through highly vascularized membranes such as the meninges which can lead to cerebral hemorrhage.

### What is required to prevent anthrax toxemia?

It is assumed that vaccination with PA-containing vaccines or immunotherapy with antibody to PA, work to prevent PA binding to host cell receptors and the downstream toxic sequelae. PA has been co-crystallized with CMG2 and the binding of PA to this receptor and to TEM8 has been characterized extensively (van der Goot and Young, 2009). Evidence that preventing PA binding to cell surface receptors is sufficient to inhibit cytotoxicity, without needing to prevent internalization of the PA^7mer^ comes from microelectrode array studies (Tournier et al., 2009). A biocompatible microelectrode array, coated with J774 mouse macrophages, has been used to investigate the cellular effects of PA binding (Trouillon et al., 2012). It was found that exposing macrophages to 20 μg.ml^−1^ of rPA activated the inducible isoform of NO synthase (iNOS), thus increasing the extracellular concentration of NO and nitrite, in a dose- and time-dependent manner. The induction of iNOS in J774 cells could be detected with rPA concentrations as low as 10 ng.ml^−1^ if the cells were pre-treated with the pro-oxidant azidothymidine. Interestingly the binding of domain 4 of rPA (PA4) alone to cells, involving an intracellular cascade through the ERK 1/2 and the PI-3/Akt kinase pathways, was as effective in triggering this response. Inhibition of either intracellular pathway abrogated the NO response. Furthermore, pre-treatment of the cells with antibody to PA or to PA4 also abrogated this effect, demonstrating that it was specific. Thus, high affinity antibody specific for PA, administered as an immunotherapy (Albrecht et al., 2007; Riddle et al., 2011) or circulating antibody which has been induced by vaccination with PA-containing vaccines, should block PA binding to the CMG2 or TEM8 receptors. A recent study has shown that monocytes from human subjects who had recovered from cutaneous anthrax, had reduced expression of the TEM8 receptor, despite enhanced IFNγ recall responses to PA by their PBMCs (Ingram et al., 2013).

This is not to say, of course that a cell-mediated immune response is not required for protection against anthrax infection. Extensive studies with PA-containing vaccines in animals and in man have shown that PA induces a strong and specific Th (CD4^+^)- mediated response in vaccinees, whose polarization will be strongly influenced by the formulation, particularly the adjuvants used (Flick-Smith et al., 2002a; Boyaka et al., 2003; Bielinska et al., 2007; Park et al., 2008; Ingram et al., 2013). This cellular memory response sustains titres of PA-specific antibodies.

### Structure: Function activity in PA

The crystal structure of the 83 kDa PA protein has been solved (Petosa et al., 1997) and shows that PA comprises four distinct domains, with domain 1a being removed by furin cleavage at the host cell receptor surface. Subsequently PA undergoes a conformational rearrangement to form PA^7mer^ and LF/EF bind sites located in domain 1b and the adjacent domain 3. Upon activation of an acidic switch, a long loop from each of the seven PA monomers rearranges into a β-hairpin forming a 14-stranded transmembrane porin-like β barrel, through which LF (and probably EF) are translocated in unfolded form into the host cell where they re-fold (reviewed in Abrami et al., 2013). This rearrangement means that domain 4 is the principal contact with the host cell receptor. A study to determine the relative immunogenicity of recombinant proteins representing these domains of PA was undertaken in a murine model of anthrax infection (Flick-Smith et al., 2002b). Whilst all the domains were immunogenic, only immunization with domain 4 protected mice against challenge with *B. anthracis*, and to the same level as that provided by intact PA. PA domain 4 contains the host cell binding region (Little et al., 1996) and is critical for the function of PA as a pore-forming toxin (Lacy et al., 2004).

### Structure: Function activity in LF

LF is a protein of similar mass to PA, which also comprises four distinct domains: an N-terminal PA-binding domain 1; domain 2 involved in binding to residues in MEK substrates distant form the cleavage site; a small domain 3 involved in binding to the cleavage site of MEK substrates and a catalytic domain 4. Domains 3 and 4 are together required for binding MEK substrates, to achieve cleavage (Ascough et al., 2012). As for PA, the domains of LF have been individually expressed as recombinant proteins and used to probe sera from volunteers immunized with the AVP vaccine for reactivity, whilst also being used to determine their efficacy as immunogens in mice (Baillie et al., 2010). Four of four vaccinated subjects recognized both PA and LF by Western blotting and all subjects had a memory response for LF, whilst two of four had a recall response to PA. Mice immunized with either LF domain 1, or intact LF were fully protected against bacterial challenge. Efficacy against a doubled challenge level was further enhanced by using a fusion of LF domain1 to PA domain 4 to immunize mice, indicating synergy between these individual protein domains (Baillie et al., 2010).

### Structure: Function activity in EF

Like PA and LF, EF is a protein of mass around 80 kDa and is a highly active calmodulin-dependent adenylate cyclase. It comprises three domains: an N-terminal binding domain 1; a catalytic domain consisting of two sub-domains which enclose the catalytic site at their interface; and a C-terminal helical domain (Liu et al., 2003). In the absence of host calmodulin, the helical domain associates with the catalytic domain to block its activity, but this protection is lost on exposure to host calmodulin which binds to the N-terminal portion of the helical domain, thus exposing the catalytic domain. Because of its localisation *in vivo* to perinuclear endosomes, EF causes a gradient in cAMP from the nucleus to the plasma membrane.

### What does the immune response to natural exposure tell us?

Natural exposure to *B. anthracis*, for example, by cutaneous infection, has been found to induce long-lived CD4^+^ Th1—mediated cellular memory to both PA and LF. The duration of infection correlated significantly with the development of a cellular recall response to PA, whilst the response to LF was focused on domain 4, with convalescent individuals having a significantly enhanced recall response to domain 4, compared with subjects previously vaccinated with AVP (Ingram et al., 2010). Epitope mapping of the CD4 response to domain 4 showed a heterogeneous response across the domain 4 sequence, but with one epitope which overlapped the catalytic center of the metalloprotease being recognized predominantly by convalescent subjects and only rarely by vaccinees. This epitope was strongly HLA-DR15-resitricted. This suggests that infection has unveiled cryptic epitopes, not commonly recognized in the response to vaccination. Subsequently, a detailed study of LF epitopes determined that immunodominant T-cell epitopes in LF exist predominantly in domains 2 and 4 and are composed of residues critical for efficient catalytic activities and substrate cleavage. An immunodominant epitope in domain II was strongly recognized by multiple diverse HLA types (Ascough et al., 2014).

The observation that the T-cell recall response of convalescent individuals was predominantly focused on the LF domains responsible for MAPKKs cleavage in the host cell is significant, since the MAPK pathways are critical in the control of T-cell activation and differentiation. Thus a host response against these epitopes is necessary for defense. LT is capable of inhibiting JNK, ERK and p38-mediated T-cell proliferation and associated Th1 cytokine secretion. ET is able to act synergistically with LT on the MAPK pathways to suppress T-cell chemotaxis and block the migration of naïve and effector T-cells to infected tissues. Together with the ability of EF to increase intracellular cAMP, the combined effect of these synergistic toxins would be to polarize naïve CD4^+^T-cells to an anti-inflammatory Th2 response, which is not optimal to counter anthrax infection.

### Clinical prospects

In the currently licensed vaccines, AVA and AVP, PA is the predominant immunogen; in addition, AVA contains traces of LF but is virtually free of EF, while AVP contains some LF and traces of EF. Non-clinical data from the use of these vaccines experimentally would suggest that both AVA and AVP are efficacious in protecting animals, when given before and, within a defined time-frame, following exposure. Recent reports from substantial and systematic studies in non-human primates have identified serological correlates of protection which predict vaccine efficacy in man (Fay et al., 2012; Chen et al., 2014; Schiffer et al., 2015). In clinical use however, the AVA and AVP formulations can be reactogenic and current efforts are aimed at rationalizing the clinical dosing regimen to reduce dosing frequency (Anthrax ACIP and Vaccine Recommendations, 2001; Shepard et al., 2002; Wright et al., 2014) whilst enhancing immunogenicity by the inclusion of CpG, for example in the AVA formulation, as NuThrax (Ionin et al., 2013; Minang et al., 2014). The reported reactogenicity has been attributed to extraneous components in these formulations, and principally the presence of LF/EF, leading to efforts to develop a next generation anthrax vaccine as a more defined recombinant vaccine which can be manufactured more readily, without the need for high levels of biocontainment (Friedlander and Little, 2009).

The comprehensive understanding which has developed of the pathogenesis of anthrax infection provides a rationale for PA as the single required pivotal component of anthrax vaccines for prophylactic use. If the initial host cell entry event can blocked by adaptive immunity (vaccination) or passive immunity (immunotherapy) then the downstream bacteraemia and toxicity can be prevented.

Several recombinant vaccines in which PA is the single active component are in development [http://www.fda.gov/downloads/AdvisoryCommittees/CommitteesMeetingMaterials/BloodVaccinesandOtherBiologics/VaccinesandRelatedBiologicalProductsAdvisoryCommittee/UCM232400.pdf; http://www.hhs.gov/news/press/2015pres/03/20150323a.html; (Williamson et al., 2005; Friedlander and Little, 2009)]. These have the advantage of being defined, highly purified formulations which are enriched for PA. Additionally they can be formulated appropriately with current effective adjuvants e.g., TLR agonists and presented as liquid or lyophilised formulations, to maximize immunogenicity and efficacy.

Whilst there is a requirement for vaccines and therapies to be highly defined to meet regulatory requirements, this bears the risk that some of the apparently extraneous components in older, less defined preparations may contribute to protection. This is the context with anthrax vaccination, where in the future there may be the option of transferring from existing licensed products such as AVA/AVP, to a recombinant licensed vaccine, such as rPA. By the judicious use of synthetic adjuvants or excipients that engage TLR receptors, it is hoped that the best of both worlds can be achieved i.e., highly defined, recombinant active components which nevertheless deliver sufficient “danger” signals to the human immune system to induce a protective response. A precedent for this has been reported with the introduction of the safer, but less immunogenic acellular pertussis vaccine for whooping cough, where it is proposed that the safer, recombinant acellular vaccine formulated in alum could also be supplemented with CpG as a TLR agonist to provide the danger signal (Ross et al., 2013). In this formulation, alum would drive an IL1β/IL17 response, whilst CpG would drive an IFNy^+^ Th1 response; both Th1 and Th17 signaling are required for bacterial clearance and protective immunity. Similar considerations apply to the identification of candidate vaccines for tuberculosis which may provide more efficacious alternatives to BCG (Blankley et al., 2014).

### Post-exposure vaccination or immunotherapy of anthrax

It has been estimated that PA may be detected in blood at approximately 24–48 h after an individual has been exposed to *B. anthracis* spores (Malkevich et al., 2014). If PA is present, then it could be assumed that LF and EF will also be secreted and LF has been detected in rhesus macaque serum at 36 h post-infection, rising to high levels >1000 ng/ml by 120 h p.i. (Boyer et al., 2007, 2009, 2015). The therapy that is initiated will therefore depend on the period that has elapsed after suspected or actual exposure to *B. anthracis*.

In this context, AVA or AVP may be offered to an individual together with 60 days of antibiotic therapy (Chen et al., 2014). Following the anthrax letters in 2001, there was evidence of widespread non-compliance with the 60-day antibiotic regimen (Schiffer et al., 2015). In the future it may be possible to shorten the antibiotic course post-exposure, once there is sufficient experimental evidence that the combination with vaccination is efficacious. Non-clinical evidence indicates that an rPA vaccine administered in two rapid doses (days 0 and 7) under antibiotic cover (starting 6–12 h post-exposure to aerosolised *B. anthracis* spores) in a rabbit model of anthrax infection, provided full protection with no bacteremia in recipients (Leffel et al., 2012). By comparison, only 56% of rabbits that received antibiotics without rPA vaccine survived exposure to *B. anthracis* and 50% were bacteremic. However, this study also showed that when the rPA vaccine dose was reduced by 90%, whilst the levofloxacin dosage was maintained, survival was reduced to 89 and 11% of animals were bacteremic.

In 2012, a new immunotherapeutic, Raxibacumab from Glaxo-Smith Kline, was licensed by the FDA for the treatment of inhalational anthrax (http://www.fda.gov/NewsEvents/Newsroom/PressAnnouncements/ucm332341.htm). Raxibacumab is a humanized monoclonal antibody with specificity for PA. In a rabbit model of anthrax infection, 82% of animals treated with antibiotics and Raxibacumab survived exposure to anthrax, compared with 65% of animals receiving antibiotic treatment alone. A Biologics Licence application for another anti-PA monoclonal, Anthim from Elusys, has been filed this year.

Monoclonal antibodies for LF and PA have been evaluated in combination in a murine model of anthrax infection (Albrecht et al., 2007). The combined Mabs were administered 2.5 h prior to challenge with *B. anthracis* and each independently provided full protection, whilst also allowing recipient mice to generate toxin-neutralizing antibody titres *de novo* in response to the challenge, so that they were protected against a second challenge 21 days later, without any additional treatment. Similarly a monoclonal antibody to LF has been evaluated in a rat model of anthrax (Lim et al., 2005).

Some have recommended that a cocktail of Mabs to all the anthrax toxins plus the capsule, should be available for post-exposure therapy in order to target all the stages in the infection process (Chen et al., 2011; Ding et al., 2013) and that combinations of Mabs may provide enhanced protection (Pohl et al., 2013). Work on these combination approaches indicated that targeting several epitopes on LF is required for effective toxin neutralization (Pohl et al., 2013).

However, passive immunotherapy, although useful in a post-exposure context under antibiotic cover, will give only limited protection, dependent on the half-life of the antibody. A novel combination of immunization with dendritic cells (Mohamadzadeh et al., 2009) which had been pre-pulsed with rPA + CpG, together with conventional immunization with rPA in alum, served to significantly shorten time to immunity in a murine model and also to reduce significantly the bacterial load post-exposure (Thompson et al., 2014).

### Concluding comments

Significant progress has been made in the last 60 years, from first discovery of the toxins secreted by the bacterium, to the detailed understanding we have today of their toxigenic effects. This in turn allows for new approaches to the prophylaxis and therapy of anthrax. Much effort and resource has been invested in the prophylaxis and therapy, following recognition of anthrax as a contemporary biothreat subsequent to the anthrax letters attack in the US in 2001, as well as by sporadic cases of anthrax in heroin users in Europe. This has led in the past few years to the licensing of new immunotherapies and the progression through clinical trials of next generation vaccines. Together, these developments provide hope for accelerated progress in the future toward comprehensive prophylaxes and therapies for anthrax, which can be made consistently by recombinant expression, at the required scale and time, without the need for culture of the pathogen in biocontainment. However, in the drive for highly defined next generation vaccines, the need to provide sufficient, controlled “danger” signals in the formulation to simulate the natural response to an infection in the vaccinee, should not be forgotten. In the twenty-first century, this requirement is facilitated by the increasing availability of synthetic versions of TLR ligands.

### Conflict of interest statement

The authors declare that the research was conducted in the absence of any commercial or financial relationships that could be construed as a potential conflict of interest.
